# Golgi protein 73 versus alpha-fetoprotein as a biomarker for hepatocellular carcinoma: a diagnostic meta- analysis

**DOI:** 10.1186/1471-2407-12-17

**Published:** 2012-01-16

**Authors:** Ying Zhou, Xin Yin, Jun Ying, BoHeng Zhang

**Affiliations:** 1 Live cancer institute, Zhongshan Hospital, Fudan University, 180 Fenglin Road, Shanghai 200032, People's Republic of China; 2 Fudan University Library, 130 Dong'an Road, Shanghai 200030, People's Republic of China

## Abstract

**Backgrounds:**

There have been conflicting reports about serum golgi protein 73 (GP73) as one of the most promising serum markers for the diagnosis of hepatocellular carcinoma (HCC). This study was to make a systematic review about the diagnostic accuracy of serum GP73 versus alpha-fetoprotein (AFP) for HCC.

**Methods:**

After a systematic review of related studies, sensitivity, specificity and other measures about the accuracy of serum GP73 and AFP in the diagnosis of HCC were pooled using random-effects models. Summary receiver operating characteristic curve analysis was used to summarize the overall test performance.

**Results:**

Eight studies were included in our meta-analysis. The summary estimates for serum GP73 and AFP in diagnosing HCC in the studies included were as follows: sensitivity, 76% (95% confidence interval (CI) 51-91%) vs. 70% (47-86%); specificity, 86% (95%CI 65-95%) vs. 89% (69-96%); diagnostic odds ratio (DOR), 18.59 (95%CI 5.33-64.91) vs. 18.00(9.41-34.46); and area under sROC, 0.88 (95%CI 0.77-0.99) vs. 0.86 (95%CI 0.84-0.87).

**Conclusions:**

The current evidence indicates that serum GP73 has a comparable accuracy to AFP for the diagnosis of HCC, while the value of serum GP73 in combination with AFP for HCC detection deserves further investigation.

## Background

Hepatocellular carcinoma (HCC) is the third leading cause of cancer-related death worldwide. There were estimated 748,000 new cases of liver cancer worldwide in 2008, causing 696,000 deaths [1]. As most patients with HCC are diagnosed at an advanced stage with underlying liver dysfunction [2], the mortality rate of HCC is similar to the incidence rate. Early detection of HCC is therefore extremely important in improving the survival of patients. Alpha-fetoprotein (AFP), together with hepatic ultrasonography, is the most common marker used in clinical practice to detect HCC in cirrhotic patients, and has been considered the gold-standard serum marker for screening patients at high risk for HCC, as well as for the diagnosis and monitoring of responses to HCC treatment for over 40 years. But the clinical value of AFP is challenged in recent years due to low sensitivity and specificity [3-5]. In addition, AFP levels greater than 500 ng/ml are correlated with the tumor size: 80% of small HCC show no increase of AFP concentration [6]. Some patients with cirrhosis and/or hepatic inflammation could have an elevated level of AFP without the presence of tumors. Therefore, serum markers with better diagnostic accuracy are needed for HCC.

Golgi protein 73 (GP73, also known as Golph2), is a resident Golgi-specific membrane protein expressed by biliary epithelial cells in normal liver, and its expression is increased markedly in chronic liver diseases, especially in HCC cells [7]. There have been studies reporting the use of serum GP73 as a serum marker for HCC, but the results are heterogeneous and even conflicting. The objective of the present review was to synthesize and analyze the results from systematic selection of research papers that evaluated the diagnostic accuracy of serum GP73 by directly comparing it with AFP for the diagnosis of HCC.

## Methods

### Inclusion and exclusion criteria

Eligible studies were those original research articles that directly compared the diagnostic accuracy of GP73 test with AFP for HCC in the same patients, or randomly assigned patients to one of the tests using blood as the only sample type. Studies that evaluated serum GP73 or AFP levels by messenger RNA, DNA or DNA polymorphisms, or those without providing the sensitivity or specificity of GP73 or AFP were excluded.

Studies published in English and Chinese were included. Abstracts, letters, editorials and expert opinions, reviews without original data, case reports and studies lacking control groups were also excluded. No restriction was set on the year of publication.

### Identification of studies

A comprehensive systematic literature review of original researches studying the diagnostic accuracy of GP73 was performed searching the following electronic databases through May 2011: PUBMED, EMBASE, Chinese BioMedical Literature Database (CBM), Cochrane Database of Systematic Reviews, Cochrane Central Register of Controlled Trials, Database of Abstracts of Reviews of Effect (DARE), Health Technology Assessment Database and NHS Economic Evaluation Database (NHS-EED). In addition, references from included articles and relevant published reports were hand searched. No restriction was set on the language, study design, year of publication and publishes status. Subject headings and keywords used in the search process included the following: (1) GP73: GP73, golgi protein 73, golgi phosphoprotein 2, golgi membrane protein 1; and (2) HCC: HCC, hepatocellular carcinoma, liver cell carcinoma, hepatic cell carcinoma. The PUBMED search strategy was shown in Additional file 1. We did not use keywords or indexing terms for diagnostic test accuracy since they might miss relevant studies.

### Study selection

All the studies were reviewed by two reviewers (Zhou Y and Ying J) independently based on titles and abstracts, and then the full texts of potentially eligible studies were retrieved for further assessment. Disagreements between the reviewers were resolved by consensus. The authors would be contacted for further study details when necessary.

When the same author reported results obtained from the same patient population in several publications, only the most recent or the most complete report was included in the analysis to avoid overlap between cohorts.

### Data extraction

The following data were extracted from the included studies by two reviewers (Zhou Y and Yin X) independently: authors, year of publication, journal, study design, number of patients, reference test, assay type of the markers, cutoff values and raw data for the analysis of sensitivity and specificity (the number of true positive, false negative, true negative and false positive results) for comparison of patients diagnosed with HCC vs. control. Any disagreements were resolved through consultation with the third reviewer (Zhang BH).

### Assessment of methodological quality

The quality of each study was assessed according to the QUADAS (Quality Assessment of studies of Diagnostic Accuracy included in Systematic reviews) checklist recommended by Cochrane Collaboration. Each of the 11 items in the QUADAS checklist was scored as "yes", "no", or "unclear" [8].

### Representative patient spectrum

HCC typically develops in patients with chronic liver disease and cirrhosis [2]. It is in these target populations that serum markers are most urgently needed. Patients with chronic liver disease or cirrhosis who were suspected as having HCC were scored "yes". Studies that recruited healthy patients in the control group and groups known to have HCC were scored "no". Studies without sufficient information to make a judgment were scored "unclear".

### Acceptable reference standard

Histopathology is the currently acceptable reference standard recommended by EASL for HCC. If histopathology is not available, HCC diagnosis is usually established by ultrasound, MRI or CT when either of them shows a nodule with arterial hypervascularization >2 cm [9]. Studies using the reference standard consisting of the above mentioned standards were scored "yes". Studies using neither histopathology nor the above imaging modalities were scored "no". Studies without sufficient information were scored "unclear".

### Suitable time between reference standard and index test

Blood samples collected before intervention were considered acceptable, knowing that HCC is unlikely to disappear spontaneously. Therefore, studies in which blood samples were collected before intervention were scored "yes". Studies in which blood samples were collected after intervention were scored "no". Studies without sufficient information were scored the study as "unclear".

### Sample verification by reference standard

Studies in which all the patients received GP73 and AFP tests and whose disease status was confirmed by the above reference standard were scored "yes". Studies in which some patients missed the above reference standard were scored "no".

### Consistency of reference standard

Studies in which the HCC diagnosis was confirmed by the same type of reference standard (histopathology or imaging techniques) in all patients were scored "yes". Studies in which the HCC diagnosis of some patients was confirmed by histopathology while that of some other patients was confirmed by imaging modalities were scored "no". Studies without sufficient information to make a conclusive judgment were scored "unclear".

### Reference standard independent of index test

Studies that did not include GP73 and AFP in the reference standard were scored "yes", and studies that included GP73 and AFP in the reference standard were scored "no".

### Reference standard blinded

Studies in which blood samples for GP73 and AFP measurement were analyzed by technicians who were blind to the reference standard results were scored "yes". Studies in which blood samples for GP73 and AFP measurement were analyzed by technicians who were aware of the reference standard results were scored "no". Studies without providing sufficient information were scored "unclear".

### Index test blinded

Studies in which the disease status was confirmed by the reference standard in all patients without the results of GP73 and AFP were scored "yes". Studies with known GP73 or AFP results were scored "no".

### Relevant clinical information

Studies were scored "yes" in which the same clinical data were available when test results were interpreted as would be available when the test is used in practice. Studies with unavailable clinical data in practice were scored "no". Studies without sufficient information were scored "unclear".

### Uninterpretable/intermediate test results reported

Studies in which all uninterpretable or intermediate results were reported were scored "yes". Studies without reporting intermediate results were scored "no". Studies without sufficient information to judge were scored "unclear".

### Explained withdrawals

Studies in which all details that happened to the patients included were clearly reported were scored "yes". Studies without explaining the reason for withdrawal were scored "no".

### Data analysis

Using the 'metandi' module for Stata (version 10), sensitivity and specificity were calculated, and the diagnostic accuracy was summarized for each study. Meta-regression was performed in an attempt to explain the observed heterogeneity. Data were presented as forest plots and receiver operating characteristic curves. Forest plots display the sensitivity and specificity of individual studies with the corresponding 95% confidence intervals. The receiver operating characteristic curves show individual study data points with size proportional to study weight, and the hierarchical summary curve resulting from the hierarchical summary receiver operating characteristic model.

## Results

**Figure 1:**
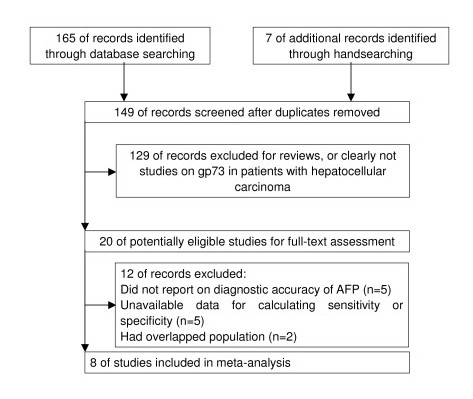
**Flow diagram indicating the process of selecting articles for meta-analysis**.

A total of 149 articles were found, of which 20 publications dealing with GP73 for the diagnosis of HCC were considered to be eligible for inclusion in the analysis. After full-text review, 12 studies were excluded: five articles were excluded because they did not allow the calculation of sensitivity or specificity [10-14]; five articles were excluded because of lacking AFP test [7,15-18], and two were excluded because they had a suspected overlap study population with another study in which results were most completely reported (Table 1) [19,20]. Finally, 8 studies were available for the meta-analysis, including 5,988 patients who received both serum GP73 and AFP tests (Figure 1) [21-28]. The characteristics of each study are shown in Table 2.

**Table 1:** Characteristics of the excluded studies

study	Reason for exclusion
Block 2005	Patients: HCC vs. Healthy Tests: immunoblot assay for GP73, AFP not measured. Outcomes: no usable data.

Mao 2008	Suspected overlapped study population with Mao 2010

Chen 2011	Suspected overlapped study population with Mao 2010.

Li 2009a	Patients: HCC vs. cholangiocellular carcinoma Tests: GP73, AFP and VEGF expression in tumor, tumor-adjacent and normal liver specimens by immunohistochemistry. Outcomes: no usable data.

Riener 2009a	Patients: HCC vs. (healthy and viral hepatitis) Tests: methods for GP73and AFP test not cleared reported Outcomes: no usable data.

Riener 2009b	Patients: HCC vs. (chronic HCV infection, bile duct carcinoma and healthy) Test: sera GOLPH2 detection using ELISA, AFP not tested. Outcomes: no usable data.

Stenner 2009	Patients: HCC vs. (HCV, bile duct carcinoma and healthy) Tests: sera GOLPH2 levels by ELISA, unclear whether AFP tested or not. Abstract; no clear data reported.

Li 2009b	Patients: HCC vs. (cirrhosis and healthy) Tests: methods for serum GOLPH2 and AFP not reported. Letter; no usable data.

Yamamoto 2009	Patients: HCC Tests: serum GP73 autoantigen/autoantibody and AFP measured Abstract, no usable data.

Yamamoto 2010	Patients: HCC Tests: serum GP 73 autoantigen and autoantibody measured by ELISA. Outcomes: sensitivity/specificity at cutoff points was not calculated.

Tan 2009	Patients: HCC vs. (liver disease without HCC and healthy) Tests: serum GOLPH2 levels by ELISA, AFP not measured. Outcomes: sensitivity, specificity and cut off value were available, but AFP levels were not tested.

Gu 2009	Patients: HCC vs. cirrhosis Tests: GP73 by ELISA, AFP not tested. Outcomes: sensitivity, specificity and cut off value were available, but AFP levels were not tested.

**Table 2:** Characteristics of included studies

study	country	HCC/controls	tumor staging (TNM)	GP 73		AFP	
				
				assay type	cutoff value	assay type	cutoff value
Hu2009	China	31/93	NK^b^	westernblot	7.4 RU^a^	ELISA	36 ug/L

Morota2011	USA	70/156	NK	ELISA	94.7 ug/L	ELISA	15.3 ug/L

Ozkan2010	Turkey	75/83	3/20/14/38	ELISA	2.36 ug/L	ELISA	4.36 ug/L

Mao2010	China, USA	789/3428	NK	immunoblot	8.5 RU	ELISA	35 ug/L

Marrero2005	USA	144/108	17/52/59/16	immunoblot	10 RU	ELISA	99 ug/L

Tian2010	China	153/219	23/95/30/5	ELISA	113.8 ug/L	ELISA	13.6 ug/L

Wang2009	USA	164/113	38/70/34/22	immunoblot	NK	ELISA	NK

Shi2011	China	55/107	NK	ELISA	100 ug/L	ELISA	400 ug/L

a Relative Unit; b Not Known

### Quality of the studies

**Figure 2:**
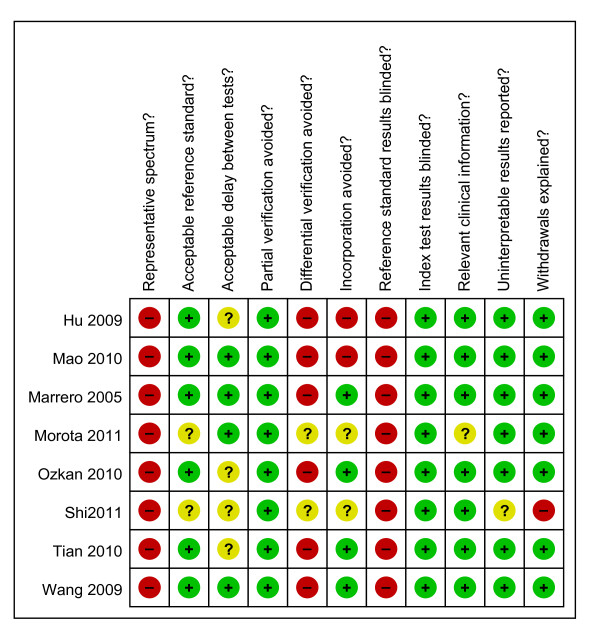
**Summary of methodological quality of included studies on the basis of review authors' judgments on the 11 items of QUADAS checklist for each study**.

QUADAS quality assessment of the included studies is shown in Figure 2. Summary scores were not calculated because their interpretation is problematic and potentially misleading [29]. However, the quality was not satisfactory. All studies used a retrospective design, and in three studies the blood samples were collected from consecutive patients. Seven studies recruited healthy people in the control group. The percentage of HCC in these studies ranged from 18.7% to 59.2%. Six studies reported the diagnostic standard of HCC, and four reported the tumor stage of the cancer patients included. Three studies clearly stated that blood samples were collected before any intervention but none of the eight studies interpreted serum GP73 test levels with diagnosis blinded.

### The summary diagnostic accuracy of GP73 vs. AFP for HCC

**Figure 3:**
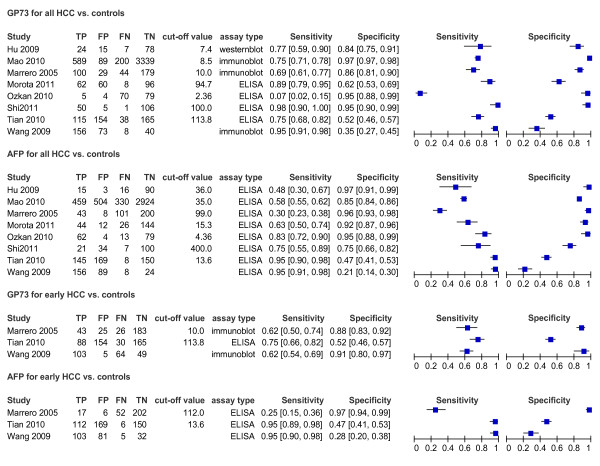
**Forest plot of pairs of sensitivity and specificity in each study included**. TP = true positive; FP = false positive; FN = false negative; TN = true negative. Forest plots document the extracted data for each study together with estimates of sensitivity and specificity accompanied by 95% CIs.

The sensitivity that these studies observed ranged from 6.7% to 90.9% (summary 76%; 95%CI 51-91%), 30% to 95.2% (summary 70%; 95% CI 47-86%) for GP73 and AFP levels in the diagnosis of HCC, respectively, while the specificity ranged from 51.8% to 97.4% (summary 86%; 95%CI 65-95%), 47.1% to 99.1% (summary 89%; 95%CI 69-96%) (Table 3). We also noted that the summary diagnostic odds ratio (DOR) and the area under sROC was 18.59 (95%CI 5.33-64.91) and 0.88(95%CI 0.77-0.99) versus 18.00 (95%CI 9.41-34.46) and 0.86(95%CI 0.84-0.87) for GP73 and AFP respectively. We presented the paired sensitivity and specificity with 95%CI of each study in the forest plot, and the significant heterogeneity was observed (Figure 3).

**Table 3:** Summary of diagnostic accuracy of GP73 and AFP using "metandi" module in stata10

summary	GP73			AFP		
	
	**Coef**.	Std. err	95% CI	**Coef**.	std err	95% CI
sensitivity	0.76	0.10	0.51-0.91	0.70	0.10	0.47-0.86

specificity	0.86	0.07	0.65-0.95	0.89	0.06	0.69-0.96

DOR	18.59	11.86	5.33-64.91	18.00	5.96	9.41-34.46

**Figure 4:**
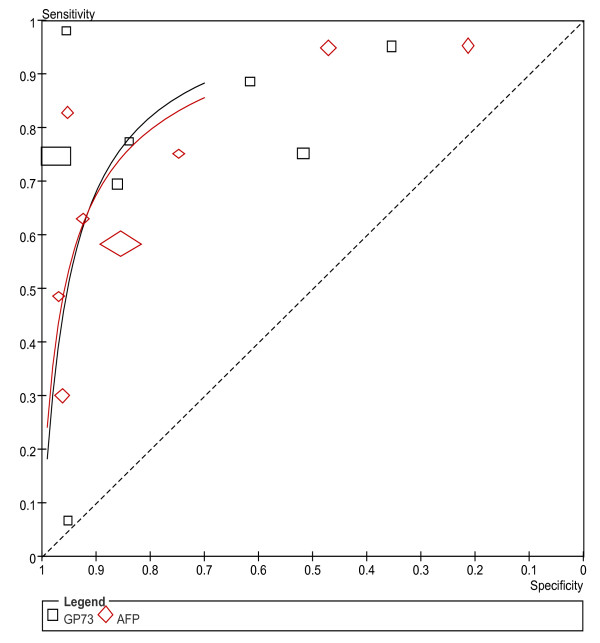
**Summary receiver-operating characteristic curves for GP73 and AFP from the hierarchical summary receiver operating characteristic model**. Circle indicates included studies, with the size of each study. The dashed lines link together the GP 73 and AFP results from each study. The curves indicated that GP 73 is comparable to AFP as diagnostic marker for HCC.

The SROC approach is the standard method for meta-analyzing diagnostic reporting pairs of sensitivity and specificity [30]. It uses DOR as the main outcome measure which removes the effect of a possible threshold [31]. So we presented the sROC curves obtained by using the parameters of the hierarchical model to present overall summary of GP73 and AFP. As is shown in Figure 4, the SROC curve of paired data of serum GP73 and AFP indicates that the GP73 curve falls near the AFP curve. Thus, we conclude that serum GP73 had a comparable accuracy to AFP as a diagnostic marker for HCC. Because the threshold level varies from study to study, we did not estimate the summary points and 95% confidence region for the two markers.

### The diagnostic accuracy of GP73 vs. AFP for early HCC

The diagnostic values of GP73 vs. AFP for detecting early HCC (TNM I-II) were reported in three studies. The raw data for the two markers are shown in Figure 3. As the "metandi" command in Stata requires data from more than four studies, we used the Metadisc (Version 1.4; Clinical Biostatistics Unit, Ramon y Cajal Hospital, Madrid, Spain). The pooled sensitivity and specificity for GP73 were comparable to AFP: sensitivity 79% (95% CI 74-84%) vs. 79% (74-83%) and specificity 62% (95% CI 58-66%) vs. 60% (56-64%). While DOR turned to be better for AFP than GP73: 11.85 (95% CI 6.92-20.30) vs. 8.03 (95% CI 2.73-23.58).

### Meta- regression for heterogeneity

The heterogeneity presented within the studies can be also observed in the SROC diagram. We attempted to explain this heterogeneity by exploring the study characteristics using meta-regression. The accuracy measure used was DOR, as it is a unitary measure of diagnostic performance that encompasses both sensitivity and specificity or both LR positive and LR negative. But limited by the small number of studies, we found that the differences for prevalence of HCC, cut-off value, consecutive patient recruitment, and assay methods used for markers did not have a statistically significant effect on DOR (Table 4).

**Table 4:** Meta-regression of the effects of methodological characteristics on diagnostic accuracy

logDOR	GP73			AFP		
	
	**Coef**.	*p *value	adjusted p*	**Coef**.	*p *value	adjusted p
prevalence	0.68	0.84	1.00	-1.02	0.79	0.98

cutoff	-0.01	0.82	0.99	-0.03	0.54	0.86

assay	0.03	0.99	1.00	-	-	-

consecutive	-2.49	0.37	0.72	0.78	0.45	0.81

*adjusted *p*-value results from permutation (20000) model in stata10

## Discussion

We found and assessed eight studies that directly compared the diagnostic accuracy of serum GP73 and AFP in the same patient population. The results demonstrate that GP73 is a comparable marker for HCC to AFP. However, the studies showed numerous methodological limitations, a broad range of diagnostic accuracy values and heterogeneity. The methodological quality of the eight studies is poor, and one of the studies did not even report the age and sex of the patients included. Five of the studies stated that serum GP73 was advantageous over AFP, while the remaining three studies reported opposite results. Because of the limited number and the different cutoff values of the studies included in this meta-analysis, we were not able to explore causes for the existing big heterogeneity by meta-regression.

The increasing incidence of HCC worldwide has sparked a new interest in HCC serum markers. A number of novel candidate markers have been suggested. GP73 is a 400-amino acid 73-kDa transmembrane glycoprotein that normally resides in epithelial cells of many human tissues [32]. Higher levels of serum GP73 were first found in patients with hepatitis B virus-related HCC by Block in 2005 [7]. Although the precise mechanism of GP73 elevation in the circulation remains obscure, serum GP73 has gained great interest for its potent role in the diagnosis of HCC. Western blotting, immunoblotting and ELISA are three major methods used to assay GP73. The former two are semiquantitative and laborious, but the results of ELISA are disappointing. Six studies [18,21,23,24,27,33] using ELISA method failed to find significant elevation of serum GP73 in HCC groups compared with that in liver cirrhosis groups. Recently described GP73-specific serum autoantibodies might interfere with ELISA analysis. Researchers have found several isoforms of GP73 that correspond with different patterns or levels of glycosylation [34]. Whether measuring HCC-specific GP73 isoform helps improve the diagnostic accuracy still needs further research.

The goal of cancer-screening is to detect tumors at an early stage when successful treatment is possible [35]. An ideal biomarker should be specific and noninvasive. The field of HCC serum markers is primed for new candidate markers that have the potential to replace their flawed leader AFP [36]. To avoid substantial confounding factors in comparisons, we only used studies that directly compared GP73 with AFP in the same patients. However, fully paired studies are usually scant. Despite the big heterogeneity, we still found that GP73 may be a comparable marker to AFP for HCC. However, cancer is a diverse class of diseases that differ widely in their causes and biology, and therefore it is unlikely for a single biomarker to detect all cancers of a particular organ with high specificity and sensitivity. The diagnostic value of GP73 combined with AFP for HCC was stated in Wang's and Mao's reports, and the results seem better than the single marker. However, further observation is needed in more studies.

Only three studies reported diagnostic utility of serum GP73 compared AFP by tumor stage and the conclusions were conflicting. The diagnostic ability of GP73 to detect HCC in an early stage needs further observation.

One of the major goals of meta-analysis is to explore reasons for heterogeneity rather than computation of a single summary measure [37]. Notably, the differential cutoff values within the studies could cause a threshold effect. Different study populations appeared to have substantially influenced the diagnostic sensitivity [38]. In our meta-analysis, the prevalence of HCC, cutoff value, consecutive patient selection and assay method were used in the meta-regression analysis to assess the effect of study methodology and threshold on DOR. Because of the small number of studies included, we used the permutation test recommended by Higgins and Thompson [39]. But we failed to find the reason responsible for the existing heterogeneity. The following reasons may hamper the statistical analysis of sources of heterogeneity: First, many reports of studies on diagnostic accuracy lacked information on key elements of design and conduct. Without complete and accurate reporting, we cannot correctly identify potential sources of bias and variability. Second, few studies using direct comparisons are available. The small numbers also made subgroup analysis unavailable. Third, the diagnostic meta-analysis is also threatened by publication bias. Investigating publication bias for diagnostic tests is problematic. Funnel plot-based tests used to detect publication bias in review of randomized controlled trials have proven to be seriously misleading for diagnostic studies, and alternatives have poor power [40].

## Conclusions

In conclusion, our meta-analysis found that GP73 is a comparable marker to AFP as an independent diagnostic tool for the diagnosis of HCC. The combination value of GP73 and AFP still needs further research. The currently existing studies on GP73 have not yet met the most stringent criteria defined by the Early Detection Research Network. Future studies should be designed to prospectively test markers in appropriately selected risk populations.

## Competing interests

The authors declare that they have no competing interests.

## Authors' contributions

ZY and YJ conceived and designed the study; searched and acquired the data; ZY and YX analyzed, and interpreted the data; ZY drafted the manuscript. ZBH analyzed and interpreted the data, provided statistical expertise, and critically revised the manuscript. All authors had full access to all of the data (including statistical reports and tables) in the study and can take responsibility for the integrity of the data and the accuracy of the data analysis. All authors read and approved the final manuscript.

## Pre-publication history

The pre-publication history for this paper can be accessed here:

http://www.biomedcentral.com/1471-2407/12/17/prepub

## Supplementary Material

Additional file 1 **PUBMED search strategy**. Click here for file
