# Supplementary material for: Golgi protein 73 versus alpha-fetoprotein as a biomarker for hepatocellular carcinoma: a diagnostic meta- analysis
Source: BMC Cancer. 2012 Jan 16;12:17. doi: 10.1186/1471-2407-12-17 (PMC3292967; doi:10.1186/1471-2407-12-17)
Supplement: Additional file 1 — PUBMED search strategy. [file 1471-2407-12-17-S1.DOC]

PUBMED search strategy：

#1 GP73

#2 golgi protein 73

#3 golgi phosphoprotein 2

#4 golgi membrane protein 1

#5 #1 OR #2 OR #3 OR #4

#6 Carcinoma, Hepatocellular [MESH]

#7 HCC

#8 hepatocellular carcinoma

#9 liver cell carcinoma

#10 hepatic cell carcinoma

#11 #6 OR #7 OR #8 OR #9 OR #10

#12 #5 AND #11
